# Development of a Finite Element Head Model for the Study of Impact Head Injury

**DOI:** 10.1155/2014/408278

**Published:** 2014-10-22

**Authors:** Bin Yang, Kwong-Ming Tse, Ning Chen, Long-Bin Tan, Qing-Qian Zheng, Hui-Min Yang, Min Hu, Gang Pan, Heow-Pueh Lee

**Affiliations:** ^1^College of Automobile and Traffic Engineering, Nanjing Forestry University, Nanjing 210037, China; ^2^Department of Mechanical Engineering, National University of Singapore, Singapore 117576

## Abstract

This study is aimed at developing a high quality, validated finite element (FE) human head model for traumatic brain injuries (TBI) prediction and prevention during vehicle collisions. The geometry of the FE model was based on computed tomography (CT) and magnetic resonance imaging (MRI) scans of a volunteer close to the anthropometry of a 50th percentile male. The material and structural properties were selected based on a synthesis of current knowledge of the constitutive models for each tissue. The cerebrospinal fluid (CSF) was simulated explicitly as a hydrostatic fluid by using a surface-based fluid modeling method. The model was validated in the loading condition observed in frontal impact vehicle collision. These validations include the intracranial pressure (ICP), brain motion, impact force and intracranial acceleration response, maximum von Mises stress in the brain, and maximum principal stress in the skull. Overall results obtained in the validation indicated improved biofidelity relative to previous FE models, and the change in the maximum von Mises in the brain is mainly caused by the improvement of the CSF simulation. The model may be used for improving the current injury criteria of the brain and anthropometric test devices.

## 1. Introduction

Traumatic brain injuries (TBI) are a great burden for the society worldwide; for example, in the US, there are about 1.4 million people who sustained TBI each year and estimated one-fifth of the hospitalized persons cannot return to work [1]. In the UK, TBI accounts for 15–20% of deaths between the age of 5 and 35 years [2]. Similar result was shown in studies made in France [3]. China is one of the countries with highest number of traffic fatalities in the world. Biomechanical study of TBI is still in initial stage [4]. To develop a better understanding of crash-induced injuries required in designing injury countermeasure, several experimental and numerical approaches have been applied [5]. Experimental approaches have been used to replicate collision damage in lab conditions using postmortem human subjects (PMHS) impact devices [6]. However, understanding the TBI mechanisms is challenging owing to inherent variation in regard to PMHS material properties and anthropometry. With rapid increases in computational technology, several human numerical models have been developed for vehicle safety research [7]. The human finite element (FE) models of the head are nowadays the most sophisticated numerical models, which can provide general kinematics of the brain and calculate the detailed strain/stress distributions which can be correlated with the risk of head injuries. Although the FE modeling of human head has been advancing over the past few decades, there is still a requirement for further research on the explanation of head injury mechanisms and for the exploration of various head protective equipment.

While several FE head models have been developed to investigate traffic accidents involving occupants in vehicles and pedestrians [8–11]. These FE head models, spanning from simple spherical shape 2-dimension models to complex 3-dimension models, have proven to be helpful tools to predict head acceleration responses which were hard to be evaluated experimentally. However, most of the FE models were built to measure a specific aspect of head injury. Among all the FE human head models in the published literature, only several complicated models were validated against both brain motion data and brain pressure [12, 13]. Among these studies, Mao et al. [13] investigated most of the head responses including the brain pressure, relative skull-brain motion, skull response, and facial response except for bridging vein stretch. Considering that head injury can be complex in the event of vehicle collision accidents with multiple symptoms on skull, face, or intracranial structures owing to various injury mechanisms, it is necessary to develop a numerical head model which is able to accurately predict a complete set of head responses.

Furthermore, most previous FE models have been validated against ICP experiments but it has been shown that a correct pressure response in the brain does not necessarily mean that the prediction of the strain is correct [6, 14]. Also, many existing models assumed either simplified material models (linear elasticity), idealized geometries, or geometries of a 50th percentile male human head. It is suggested that the size of the head [15], the skull-brain boundary condition [6, 16], such as the central fissure and sulcus, or the suspension system, such as the tentorium and dura mater [17, 18], can change the mechanical response of the brain. It can be seen that the shape of the skull, the composition of gray and white matter, and geometry of other soft tissues and the volume of cerebrospinal fluid vary greatly between even similar sized tissues. This suggests that the accurate FE head models need to be developed to enhance their biofidelity.

Based on the above considerations, the purpose of this study is to develop a more biofidelic FE human head model using the geometry directly reconstructed from the medical scan data of a 50th percentile male volunteer. Such an FE head model should mimic irregular anatomic features of the head, is validated against a full spectrum of head impact data, and can be applied in a wide range of impact situations to predict skull, facial, and intracranial responses. Therefore, it would be desirable to include those anatomical structures that will improve quality and accuracy of such analyses.

## 2. Materials and Methods

### 2.1. Mesh Development

**Figure 1:**
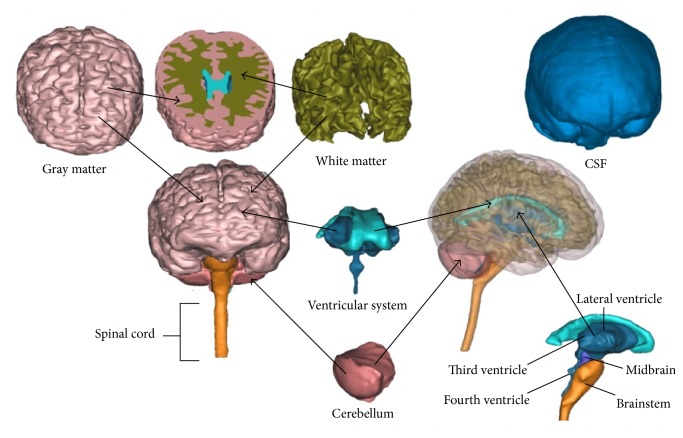
Various components in brain model.

At present, there are no FE head models suited to the characters of the Chinese on the injury mechanism of the TBI. The geometry reconstruction of the human head was conducted by the Center for Application Biomechanics, National University of Singapore [5]. A male volunteer with anthropometric characteristics close to the 50th percentile Singapore Chinese male (175.3 cm/78.2 kg, Hybrid III dummy) was recruited to develop an extensive image dataset. The resolution/thickness of the computed tomography (CT) and magnetic resonance imaging (MRI) scans were 0.488/1.0 mm and 0.500/4.0 mm, respectively. The geometries of the bony structures and soft tissues of the volunteer head region were reconstructed using the CT and MRI scanned images, using the segmentation method developed by Dale et al. [19] and later on by Fischl et al. [20]. With minimum manual edition, we sought to align the MRI to the CT, and registration accuracy was evaluated by performing analysis of the coordinate differences between CT and MR anatomical landmarks along the *x*-, *y*-, and *z*-axes. The human brain was segmented into cerebellum, gray and white matters, the entire ventricular system of the brain (i.e., lateral ventricles, third ventricle, interventricular foramen, cerebral aqueduct, and fourth ventricle), midbrain, and brainstem, with cerebrospinal fluid surrounding it (Figure 1).

**Figure 2:**
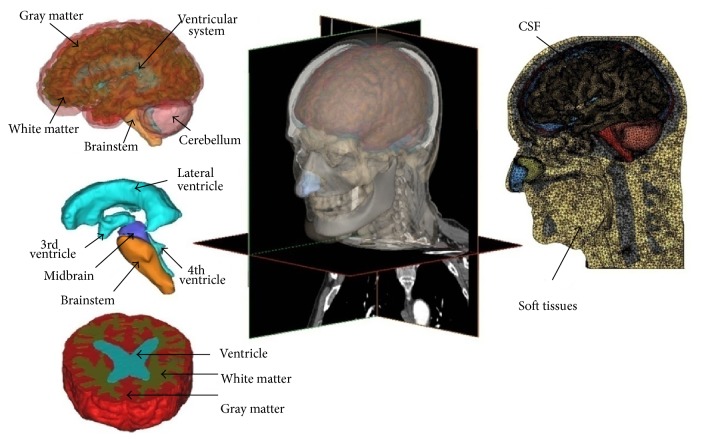
Various intracranial components of human head segmented from CT and MRI data by Mimics, which includes soft tissues as well as more detailed segmentation of brain tissue, are shown in this figure. The meshed model on the right shows the complexity of the integrated segmentation of the brain tissues.

Despite recent advancements in segmentation methods for brain tissue with magnetic resonance images (MRI) [21], there is no automatic segmentation tool available for nonbrain tissues such as extracranial tissues like cartilages, fats, and neck muscles. This was owing to the fact that segmentation of these tissue types was often ignored since these tissues were regarded as less important as compared with the skull-brain tissue and were not usually considered in the FE head model. Based on the reference to available atlas of head anatomy [22], the geometry of the cartilages, namely, the cartilage of septum and the lower and upper lateral cartilages of the human nose, is reconstructed semiautomatically using an adaptive moving mesh technique and shape preserving parameterization. The models also contain some of the interior details, which are often ignored in previous models, such as air sinuses, namely, maxillary sinuses, frontal sinus, and sphenoidal sinuses (Figure 2) [5, 23].

Multitissue mesh generation on medical images is a fundamental step for building a realistic biomechanical model. Mesh elements with large or low dihedral angles are undesirable. In the literatures, there have been studies on multitissue meshing based on Delaunay refinement [24–26]. However, elements with small dihedral angles are likely to occur in Delaunay meshes, because elements can be removed only when their radius-edge ratio is large, and their dihedral angle quality is completely ignored.

Unlike above Delaunay-based methods, Zhang et al. [27] presented a new method to generate a hexahedral and tetrahedral mesh. Firstly, this method identified the interface between different tissues and nonmanifold nodes on the boundary. Then, all tissue regions were meshed with conforming boundaries cooperatively. Finally, geometric flow schemes and edge-contraction were used to improve the quality of the tetrahedral mesh. In our work, we incorporated mesh quality, fidelity, and smoothing into one point based registration framework.

Three layers of skull hexahedral meshes were developed with HyperMesh software (Altair, Troy, MI); however, the highly folded gray and white matters had formed in an interlocking pattern. Therefore, tetrahedral elements were more preferred for discretization due to their adaptiveness to highly curved complicated structures. The existing tetrahedral meshes were optimized on combining Laplacian and optimization-based mesh smoothing, nodal points deletion and insertion, and local remeshing. The resulting meshed head model is composed of 1,173, 039 tetrahedral elements and 293, 260 nodes (Figure 2 and Table 1). For the whole head model, meshes with average edge length of about 1.57 mm and aspect ratio of 1.61 were generated.

### 2.2. Material Properties

A huge number of head material mechanical tests have been done on cadaver or animal specimens, such as mechanical stretching, indentation or shearing, compression techniques, and magnetic resonance. Summary of these material tests can be found in several review articles [28, 29]. A large range of datasets of head materials were provided in these materials studies. Combined with the large-deformation theory, linear viscoelastic material properties were assumed for the brain tissues. Skeletal tissues such as cervical cartilages and human skull were simulated as linear isotropic, elastic materials. It should be also noted that the head model involves different components and the densities of these components were adjusted to achieve the average human head weight according to a recent study by Farmanzad et al. [30].

Mechanical properties of the skull-brain interface structures are still not fully understood. Similar to but different from many other studies [31, 32], the CSF was simulated as hydrostatic fluid filled cavities with a surface-based method. The coupled structure between the pressure exerted by the contained fluid and the deformation of the fluid filled structure was defined using the surface-based method. It has an advantage over modeling fluid and the structure interaction without the need of any elements, thus preventing unreasonable distortion that could be related to an element based method. Simulating the CSF space as a number of hydrostatic fluid cavities would be desired to imitate the pressure response in CSF during a dynamic vehicle impact. The material properties of the FE model are summarized in Table 1.

## 3. Results and Discussion

**Figure 3:**
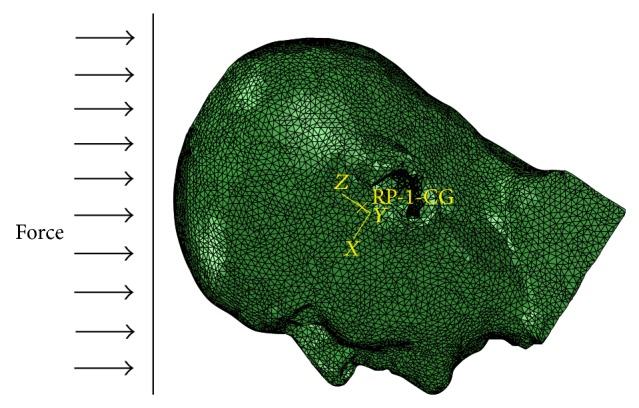
Illustration of the impact direction in numerical simulations.

To check the predictability of the FE model for the crash-related head injuries, some cases under frontal angled impact were used to validate the numerical model predicted brain pressure. In Nahum's study [33], the Frankfort anatomical plane of the head was inclined at an angle of 45 to the horizontal before impact, as illustrated in Figure 3. The impact velocities differed from 4.36 to 12.95 m/s. the acceleration from case 37 was selected as the baseline for the FE head model. A free boundary condition, which means there is no constraint effect at the head's six degrees of freedom, is used at the neck junction since Ruan et al. [34] and Willinger et al. [35] showed that the neck does not appear to influence the pressure response of the brain in short duration frontal impact (<15 ms) (in Nahum's case, the impact duration was approximately 6 ms).

The impact condition was generated by imposing force amplitude as the time history of a cylindrical impactor with impact velocity of 9.94 m*·*s^−1^ and a mass of 5.59 kg. This force amplitude curve was acquired from the impact force result reported by Nahum et al. [33]. Obviously, the largest impact force occurred at about 0.004 s. The injuries predicted by the FE model and the time history curve of impact loading were recorded and were in contrast with PMHS test data. The simulations effects of the FE model to the cadaver experiments on the impact force, intracranial pressure, the maximum von Mises stress in the brain, and the maximum principal stress in the skull will be discussed as follows.

### 3.1. Impact Force and Intracranial Acceleration Response

**Figure 4:**
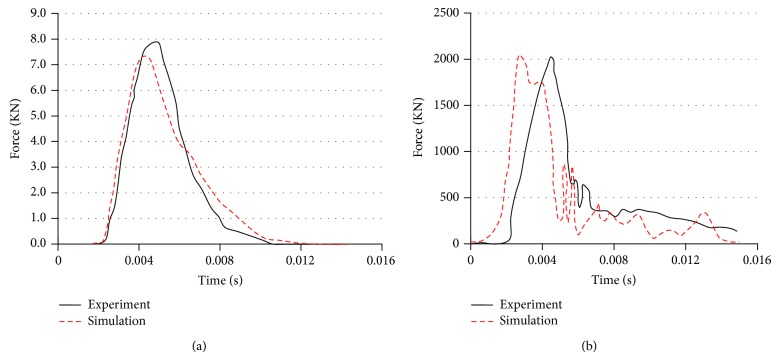
Comparison of impact force and head acceleration between simulations and the cadaver experiments. (a) Impact force; (b) head acceleration.

Under case 37 loading condition, intracranial acceleration response and the maximum impact force show random patterns, with a peak head acceleration of less than 2000 m*·*s^−2^ and a peak maximum impact force of less than 7,500 N (Figure 4). The FE head model predicts brain impact force and head acceleration curves show good agreement with those measured by experimental technique. It can be observed that the impulsive force-time graph of the head model has lower peak and longer impulse duration, which is shown in Figure 4(a). It is possible that the change in behavior is due to the “cushioning” layer of soft tissue. As seen from Figure 4(b), the calculated accelerations of the center of mass of the FE model of human head give magnitudes and characteristics similar to the experimental result. Simulation results indicate that the impulse response of the actual impact only lasts about 6 ms; therefore, it is reasonable to ignore movements of the neck to the head in a short period.

### 3.2. The Intracranial Pressure (ICP)

**Figure 5:**
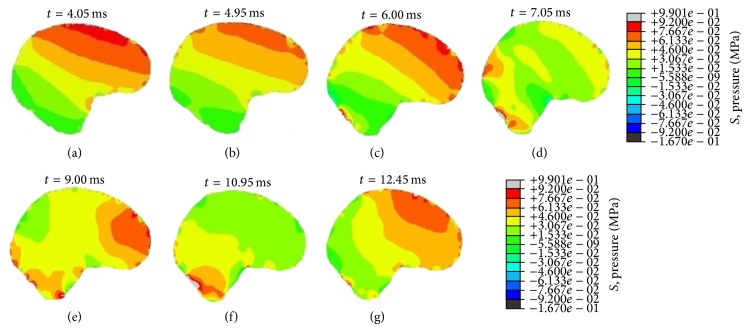
Gradient brain pressure during frontal angled impact at an angle of 45 to the horizontal.

The simulated result shows the intracranial pressure gradient generated across the brain during blunt impact (Figure 5), and the FE model predicted brain pressure agrees with those measured by Nahum et al. [33]. Stress waves that propagate in the brain are produced under rapid contact loading. Wave propagation may result in a pressure gradient with positive pressure at the site of impact (coup), negative pressure on the opposite side of the impact (contrecoup), and neutral pressure in the medium. Wave propagation of compression pressure is proposed as mechanism for the intracranial compression causing focal injuries of the brain tissue and bruising. However, it is still not fully understood whether the injury is owing to a cavitation phenomenon or owing to tensile loading (negative pressure) [36]. Furthermore, the pressure propagation can induce shear strains deep within the brain. Contact loading may also lead to a relative motion of the skull-brain surface regarding the internal aspect of the base of skull. Subdural hematoma (caused by tearing of the bridging veins) and surface contusions in the brain can be the consequences.

**Figure 6:**
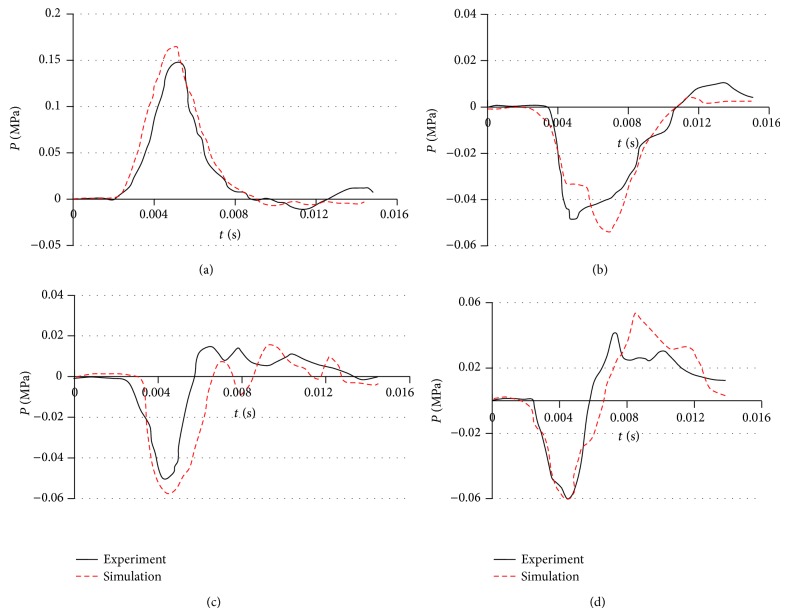
Intracranial pressure response from the FE model and cadaver experiment. (a) Frontal pressure; (b) bilateral occipital pressure (left); (c) bilateral occipital pressure (right); (d) posterior fossa pressure.

Figure 6 shows the intracranial pressure at four locations within the brain for the FE model. The trough duration in posterior fossa regions (Figure 6(d)) and bilateral occipital (Figures 6(b) and 6(c)) is more comparable with that of the experimental results, but with overestimated troughs (11.5% for posterior fossa pressure; 13.8% and 17.5% for bilateral pressures). The pressure at the coup position is the major concern in experimental head impact tests. As shown in Figures 6(a) and 6(d), the maximum pressure predicted from the FE model is 170 KPa and a minimum pressure −65 KPa. The maximum pressure at the coup position is overestimated in the FE model, and the time when the corresponding maximum pressure is reached also differs from the experimental result. These differences are due to the fact that dimensions of the head model are distinct from that of the head used in the experiments. Horgan and Gilchrist [37] reached a better agreement by modifying the dimensions of their model to match those used by Nahum et al. [33]. Generally, the occipital region experienced tension while the frontal region first experienced compression before the trend was reversed when the brain has rebounded. These indicated pressure values are actually lower than those proposed by Ward et al. [38], in which the tolerance thresholds of tension and the brain pressure for compression are, respectively, −186 KPa and 234 KPa.

### 3.3. The Maximum von Mises Stress in the Brain

**Figure 7:**
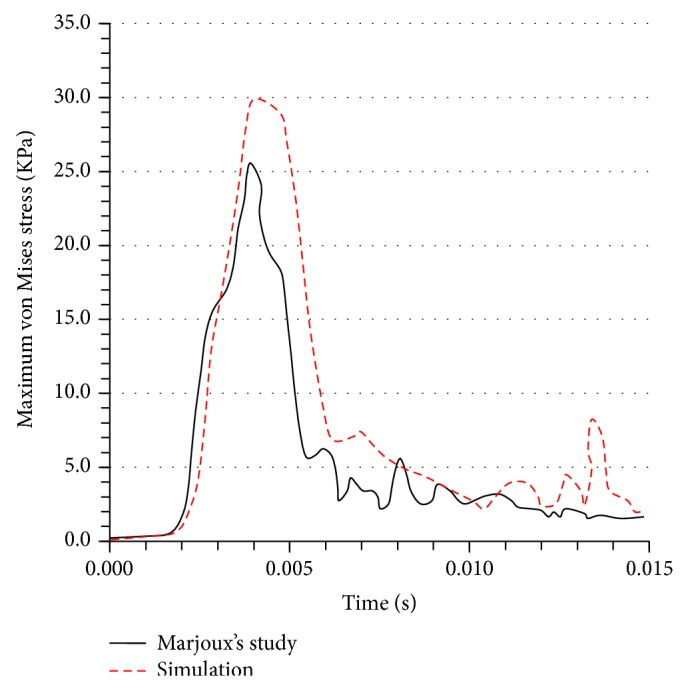
The maximum von Mises stress in the brain during the collision process.

One of the main reasons for developing an FE head model is to apply it to investigate impact head injury. The value for von Mises stress in the brain has been used to assess the risk of brain injury used by Marjoux et al. [39]. Therefore, the maximum von Mises stress in the brain during the impact simulations is shown from the FE model, as shown in Figure 7. Simulation result indicates the same trend of the maximum von Mises stress distribution as Marjoux et al.'s study during the period of impact, with the time at which the peak values occur corresponding to that of the peak impact force. However, the peak values are importantly different. It is obvious that the peak von Mises stress predicted in the FE model is approximately 18% higher than that in Marjoux et al.'s result. Therefore, when applying it to evaluate the TBI in a real collision situation, the FE model can importantly overestimate the brain damage risk [39]. In the FE head model, the CSF has been simulated as a fluid instead of a solid. Furthermore, the viscoelastic properties of the brain materials have been considered in the FE model. For these reasons, it is likely that the stresses predicted from the FE model are more accurate than those from the precious studies.

### 3.4. The Maximum Principal Stress in the Skull

With regard to skull fracture due to collision, the maximum principal stress in the skull is regarded as the suitable variable to assess the skull fracture; for example, refer to Yoganandan and Pintar [40]. In all tests, cadaver heads were mounted on individualized mold. The dynamic experiments, including four vertical impacts, one occipital impact, and one frontal angled impact, were used. The tests used a hemispherical anvil to fracture skulls with impact velocities. At the end of one frontal impact, multiple fractures at frontal bone were observed. After one occipital impact, circular fracture was observed. In four vertical impacts, fractures including linear fracture at vertex to right orbit and frontal bone; multiple skull fracture through vertex, frontal, and temporal bones; circular fracture at vertex region; and bilateral fractures at parietal bone. In simulation, the velocity and impactor were defined according to each of six experiments. The associated skull deformation and the impact were simulated.

**Figure 8:**
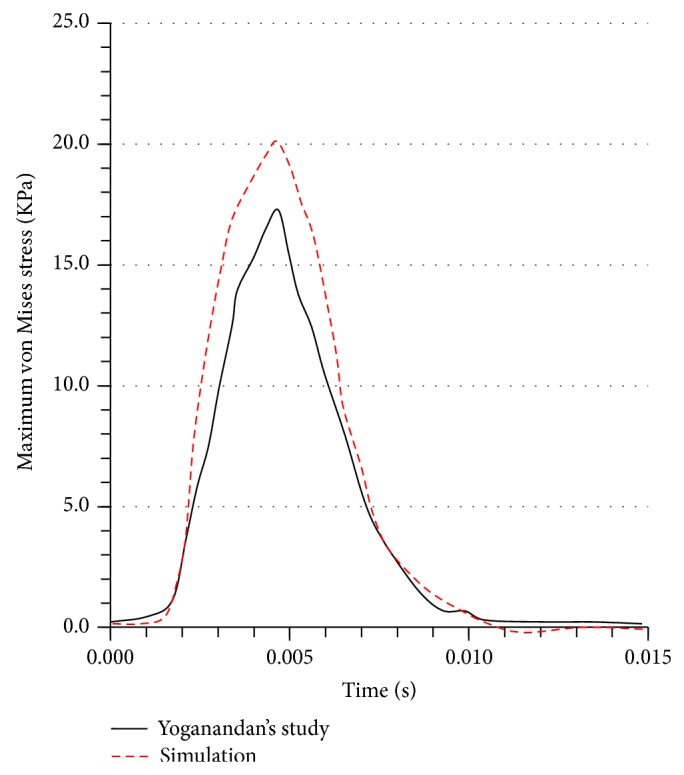
The maximum principal stress in the skull during the collision process.

Figure 8 shows the FE head model-predicted peak forces agree with Yoganandan et al.'s study. The FE model predicts frontal bone fractures, matching well with Yoganandan et al.'s data except for the zygoma injury. For occipital impact, the model-predicted force is 24% higher than the Yoganandan et al.'s result. Bone fractures happen before the reaction force reaches peak value.

### 3.5. Brain Motion

**Figure 9:**
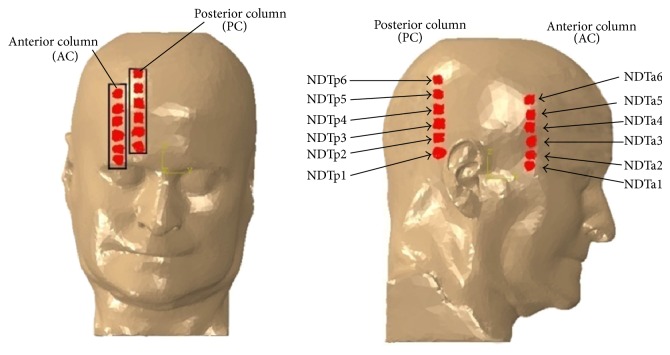
NDT column implantation configurations for head model in Hardy et al.'s [41] C383-T1 (Group A) test.

The brain motion will be validated against the experimental brain displacements with blunt impacts performed by Hardy et al. [41]. Similar to Hardy et al.'s [41] neutral-density targets (NDT) columns implantation configurations for C383-T1 test, the twelve NDTs are located in the models as shown in Figure 9.

**Figure 10:**
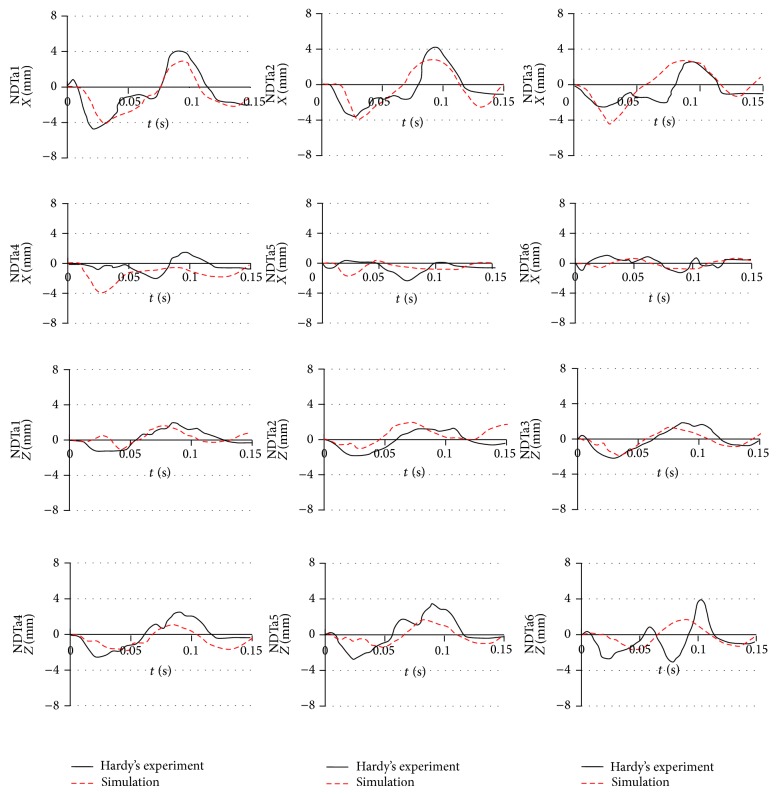
Comparison of the relative skull-brain displacement of the anterior NDTs column located in frontal lobe between that predicted by simulations of our head models and that obtained in Hardy et al.'s [41] C383-T1 frontal impact experiment of a cadaver.

**Figure 11:**
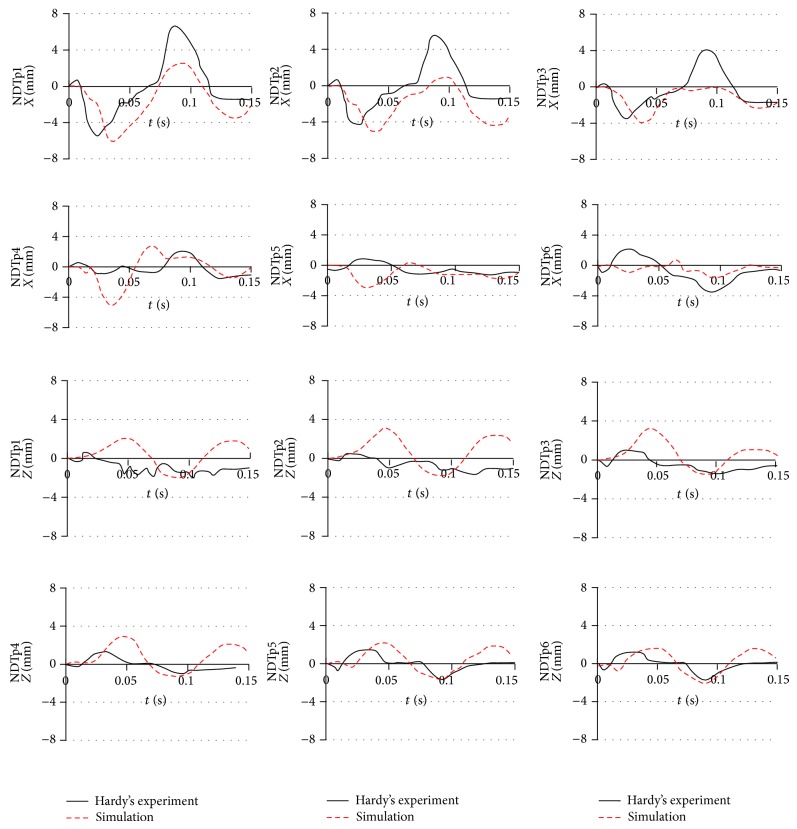
Comparison of the relative skull-brain displacement of the posterior NDTs column located in parietal lobe between that predicted by simulations of our head model and that obtained in Hardy et al.'s [41] C383-T1 frontal impact experiment of a cadaver.

The simulation results for the relative skull and brain displacement of the 2 arrays of 6 NDTs located in the parietal lobe at the right side of the head and frontal lobe are shown in Figures 10 and 11. Each plot in the figure represents the relative displacement of each NDT, which was computed using the difference of a fixed point on the skull and the absolute displacements of each NDT. The relative *x*-displacement of the NDTs in anterior column (AC) is generally characterized by a minimum occurring between 20 and 45 ms and a maximum at around 70 to 95 ms while the AC NDTs' relative *z*-displacement reaches its minimum in the range of 25 to 45 ms before restoring to the maximum in the range of 70 to 90 ms. Similar trends are acquired for the NDTs in posterior column (PC) with the minimum and maximum in *x*-direction in the range of 30 to 45 ms and 70 to 100 ms range, respectively, whist the minimum and maximum in *z*-direction are in the range of 40 to 45 ms and 80 to 95 ms, respectively.

There is an average deviation of 1.12 mm (62.83%) in the relative *x*-displacement amplitude, as well as 0.344 mm (36.76%) average difference in the *z*-relative displacement amplitude between the experiment and simulation for the head model. The simulated *x*-relative displacement amplitude deviates the most for the NDTp2 marker of head model with 4.65 mm deviation and percentage difference from experiment of 82.93%. It differs from the experiment the least in NDTa3 with 0.124 mm deviation (4.55%). The largest deviation in *z*-direction was found in NDTa5 (3.18 mm; 95.13%) whist the smallest *z*-relative displacement amplitude was located in NDTp3 (0.0475 mm; 3.41%).

When comparing the relative displacement characteristics of the simulation with the experiment, average correlation coefficient of *x*-relative displacement of 0.458 is found, while that of the relative displacement in *z*-direction is 0.516. The highest correlation coefficients of the *x*-displacement are noted in the NDTa1 marker, whereas the respective lowest *x*-displacement correlation coefficient is 0.0964.

### 3.6. Selected Future Improvement

The current head models mainly focused on the head injuries. The neck vertebrae in this work are rather simplified without further segmentation into the intervertebral disc. Furthermore, the passive and active properties of cervical musculature tissues are not modeled in the current study. A more complete head model including all the cervical ligament tissues is needed in the future to arrive at the whole picture of head and spinal injuries.

The meshing and validation of the head model can be further investigated. With increasing computing power to handle with decreasing minimum necessary time step, more detailed vasculature and brain surface shapes can be developed in the future. Regarding validation, much more loading cases will be selected in a range of experimental test data to validate more robust performance of the model predictions based on responses including brain contusion and facial response. A broken nose is one of the most common facial injuries in frontal vehicle collision. Therefore, the facial bone responses will be validated under nasal impacts performed by Nyquist et al. [42] and Allsop et al. [43]. Furthermore, the new brain injury tolerance levels are also proposed for various traumas.

## 4. Pedestrian Accident Reconstruction

Vulnerable road users—namely, pedestrians and bicyclers—often suffer severe and fatal injuries in vehicle collisions. Head injuries are a high proportion of such traffic accidents. In this study, a typical pedestrian accident is analyzed and reconstructed using the multibody pedestrian model and FE head model. The kinetic parameters and the pedestrians' postures after impact are computed based on numerical simulations. And the resulting severities of injury are investigated.

### 4.1. Collision Model Development

**Figure 12:**
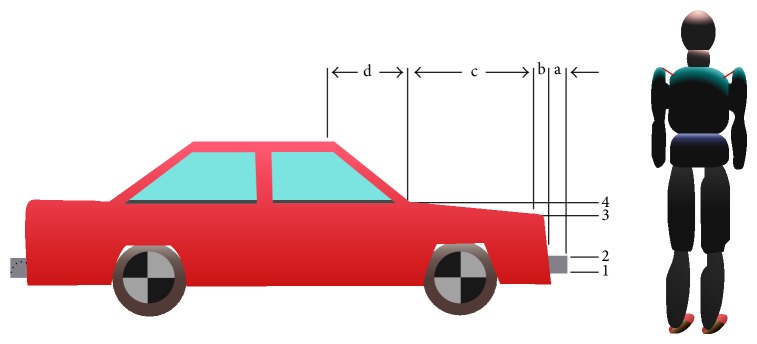
Pedestrian model and vehicle basic parameters ((1) 0.35 m; (2) 0.5 m; (3) 0.8 m; (4) 0.9 m; (a) 0.05 m; (b) 0.06 m; (c) 1.02 m; (d) 0.58 m).

This study adopts the multi-rigid-body dynamics method to investigate the vehicle, pedestrians, and pavement multi-rigid-body collision behavior. The pedestrian model adopted is a multibody dynamics system consisting of many rigid bodies. Different independent rigid body represents the various body parts of the pedestrian. For each independent rigid body, its attributes such as appearance, mass, contact rigidity, and friction factor are important parameters. The number of independent rigid bodies and hinge points influences the simulation computational time. The kinetic parameters of rigid body velocity, acceleration, and running distance can be computed in these simulations. Pedestrian model and the basic parameters of the vehicle are shown in Figure 12. The friction factor of the foot and the ground is assumed to be 0.7. The friction factor of the vehicle and the ground is assumed to be 0.67. The contact friction factor of body parts and the front vehicle is assumed to be 0.5, similar to those reported studies in [44, 45].

### 4.2. Simulation Result and Analysis

**Figure 13:**
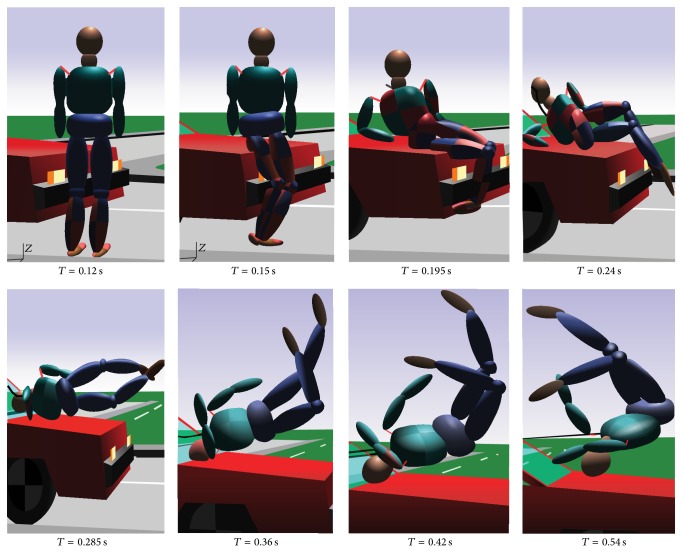
Kinematics simulation results.

The pedestrian movement process can in generally be divided into three stages such as “contact,” “post-flight flip,” and “fell to glide” after vehicle-pedestrian high-speed collision. Supposing that the vehicle velocity is 60 km/h, braking drag acceleration is exerted at the impact moment which is 0.6 g (g is the gravitational acceleration), it allows the vehicle to go straight. The direction of pedestrian's speed is perpendicular to that of the vehicle. The pedestrian passes through the road with the assumed walking speed and locates within the central vehicle area when collision occurs. Whether the vehicle is equipped with antilock braking system (ABS) or not is considered during collision simulation. The accelerations of the centre of mass (CG) of the dummy head and thoracic sides are compared under the same conditions [46]. A typical vehicle-pedestrian collision kinematics simulation results are shown in Figure 13.

The head acceleration peaks with ABS or non-ABS in the simulations are shown in Table 2. The pedestrian head injury criterion (HIC) values exhibit an increasing trend along with increased collision speed. The pedestrians HIC value does not exceed 1000 which is safety limit when the speed is lower than 60 km/h, on condition that the vehicle impacts with pedestrians' lateral body parts with low-speed after braking measures are taken, and pedestrians will not be hit in the head. Fatality is unlikely for this scenario. Consequently, when the vehicle speed exceeds 60 km/h and HIC value exceeds the safety limit, pedestrians may be multiple impacted or rolled and probably die from severe head injury.

## 5. Conclusions

A three-dimensional FE head model with detailed skull and brain structures was developed. The structural and material properties were analyzed based on a synthesis on the current state of knowledge of the tissue constitutive model. The CSF was simulated as hydrostatic fluid filled cavities based on a surface-based method. Impact force, intracranial acceleration response, intracranial pressure, brain motion, the maximum principal in the skull, and the maximum von Mises stress in the brain for short impact impulse were investigated compared to experimental head impacts. The FE head model showed good correlations with PMHS test data and precious study in terms of injury prediction and biomechanical response. When comparing the stresses in the head, the previous data underestimates the maximum von Mises stress in the brain by approximately 18%. Furthermore, it is the improvement of the CSF material's fluid properties that causes the change in the maximum von Mises stress in the brain (i.e., modeling the CSF as hydrostatic fluid cavities instead of the baseline solid material definition). Compared with diagnosis reports, this model has the capability to predict injuries while the calculated injury indices indicate a good ability to predict corresponding injury types and severity.

Despite the need for trauma reconstructions and further material properties tests, the model may be used in future for improving the current head injury criteria (HIC) and the design of anthropometric test devices (ATD). Furthermore, the present head model can be coupled together with other body regions to create a state-of-the-art human FE model to be used in the broad field of vehicle safety. It is expected the new human FE model will help in better understanding the injury mechanisms during vehicle collisions and developing advanced restraint systems.

## Figures and Tables

**Table 1:** Element number and materials properties in the FE model.

Components	Number of elements	Behaviour	Density (kg/m^3^)	Poisson's ratio	Young's modulus, *E* (GPa) Viscoelastic response (Prony series approximation)	References
Brainstem	6104	Viscoelastic	1060	0.4996	*G*(*t*) = 0.0045 + (0.0225 − 0.0045)*e* ^−*t*/80^	Horgan and Gilchrist [37]

Cerebral peduncle	1762	Viscoelastic	1060	0.4996	*G*(*t*) = 0.0045 + (0.0225 − 0.0045)*e* ^−*t*/80^	Horgan and Gilchrist [37]

Cerebellum	21727	Viscoelastic	1140	0.48	*G*(*t*) = 0.168 + (0.528 − 0.168)*e* ^−*t*/35^	Turquier et al. [47], Willinger et al. [48], Shuck and Advani [49], and Yoganandan et al. [50]

CSF	—	Fluid	1000	—	—	Zhou et al. [31] and Yan and Pangestu [32]

Gray matter	436917	Viscoelastic	1040	0.4996	*G*(*t*) = 0.0064 + (0.034 − 0.0064)*e* ^−*t*/700^	Zhang et al. [12] and Al-Bsharat et al. [51]

Lateral cartilage	2874	Elastic	1500	0.45	*E* = 0.030	Westreich et al. [36]

Septum cartilage	3578	Elastic	1500	0.32	*E* = 0.009	Grellmann et al. [52]

Skull bone and cervical vertebra	130482	Elastic	1210	0.22	*E* = 8.000	Zhang et al. [12]

Neck and facial soft tissues	253894	Elastic	1040	0.46	*E* = 0.01667	Zhang et al. [12] and Kleiven [53]

Ventricles	36776	Viscoelastic	1080	0.49	*G*(*t*) = 0.00101 + (0.101 − 0.00101)*e* ^−*t*/100^	Zhang et al. [12]

White matter	278925	Viscoelastic	1040	0.4996	*G*(*t*) = 0.0078 + (0.041 − 0.0078)*e* ^−*t*/700^	Al-Bsharat et al. [51]

**Table 2:** Head acceleration peak.

Vehicle speed (km/h)	Head linear acceleration with ABS (m/s^2^)	Head linear acceleration with non-ABS (m/s^2^)
20	340	360
25	390	480
30	260	310
35	350	350
40	380	380
45	340	330
50	490	460
55	900 (HIC = 864)	740
60	1020 (HIC = 1086)	360
